# Optimizing Acid Mist Suppression: Unraveling Surfactant Effects on Bubble Formation and Bursting Dynamics in Copper Electrowinning

**DOI:** 10.1007/s40831-025-01297-8

**Published:** 2025-10-13

**Authors:** Ashish Kakoria, Mirza Muhammad Zaid, Aamir Iqbal, Ellen Amoako Afful, Guang Xu

**Affiliations:** Department of Mining and Explosive Engineering, Missouri University of Science and Technology, Rolla, MO 65401 USA

**Keywords:** Copper electrowinning, High-speed video imaging, Air bubble, Acid mist emission

## Abstract

The working mechanism of surfactant to reduce acid mist in copper electrowinning system is not well understood. Most of the studies are based on the surface tension reduction phenomenon but this is not the only function that causes acid mist reduction. In this paper, we investigated the effect of different surfactants on a bubble’s residence time, terminal velocity, flow regime, and bursting dynamics using a high-speed camera. We have evaluated five different surfactants and found that the presence of surfactants reduces the terminal velocity, bubble diameter, and increases the residence time of the bubble in electrolyte. Especially for FC-1100, the low terminal velocity and high residence time allowed the bubble to shift its flow regime to stokes flow and allow ample time for stabilization. This was dependent on the small Weber and Ohnesorge number when FC1100 was added, which means the bubbles had less deformation and better stabilization. Therefore, the addition of FC1100 generated bubbles with the lowest kinetic energy during rupture as compared to other surfactants. Most of the kinetic energy is used to elongate the bubble film and a little kinetic energy is used to burst the bubble. To validate our study, we have performed acid mist experiments in a chamber to measure different surfactants’ suppression efficiencies. The results of the study relate terminal velocity, residence time, kinetic energy and bubble diameter to suppression efficiencies that help the understanding of surfactant’s mechanism. Compared to previous studies our work has certain novelties in understanding of bubble growth, propagation and bursting mechanism in a copper electrowinning system in the absence and presence of surfactants with emphasis on surface tension, bubble terminal velocity, residence time and Kinetic energy. While previous research has primarily examined bubble size and its influence on mist generation, as well as the role of surfactants in altering bubble size and suppressing mist, this study expands the focus to include the dynamic behavior of bubbles throughout the life cycle of the bubble.

**Graphical Abstract:**

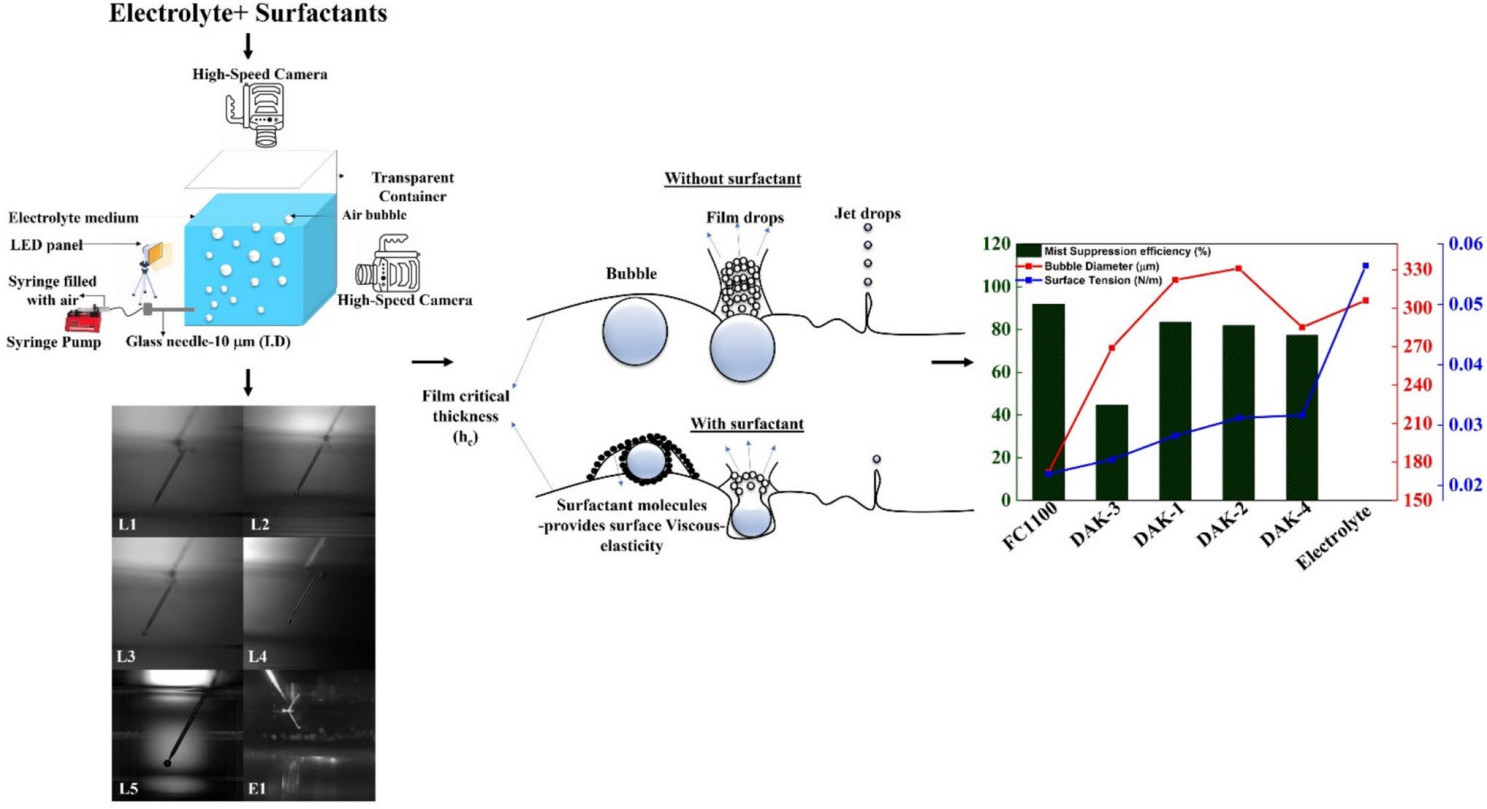

## Introduction

Acid mist generated from copper electrowinning poses serious environmental and health risks, necessitating stringent laws to preserve ecosystems and human health [1]. This mist is formed when oxygen gas, produced at the anode during the electrochemical extraction of metals from their ores in a sulfuric acid electrolyte, splashes and aerosolizes the acidic solution [2, 3]. The environmental impact includes air pollution that causes acid rain, water contamination, and soil acidification, all impairing ecosystems and biodiversity. Inhaling acid mist has serious health consequences, including respiratory problems, skin and eye irritation, and long-term lung illness. To reduce these dangers and safeguard the environment, regulatory authorities such as the Environmental Protection Agency (EPA) and the Occupational Safety and Health Administration (OSHA) have created severe emission standards and occupational exposure limits, namely EPA 450/2-77-019 (1 mg/m^3^) and 1926.55 (1 mg/m^3^) respectively [2, 4, 5].

Due to environmental concerns, the discontinuation of FC-1100 in the copper electrowinning sector has sparked the hunt for new surfactants that can effectively reduce acid mist emissions while being environmentally friendly. A few non-fluorinated surfactants, such as sodium lauryl sulfate (SLS), ethoxylated alcohols and polysorbates are the most frequent replacements [6–8]. These surfactants reduce the electrolyte’s surface tension, resulting in a stable foam layer that prevents acidic aerosols from being released. Furthermore, several silicone-based surfactants and biodegradable chemicals [9], such as alkyl polyglucosides [10], have received attention for their efficacy and reduced environmental impact [11]. While these options have various degrees of success in minimizing acid mist, further research is required to enhance their performance [12]. Finding a compromise between acid mist suppression efficacy and environmental safety remains a problem, underscoring the need for continuing surfactant chemical research [2, 13].

However, scientific research information on the mechanisms of these surfactants is needed. A few studies have been conducted to understand their mechanism by comparing the use of a surfactant with the number of bubbles generated. They observed a significant reduction in the bubble size with surfactant and a noticeable change in the burst mechanism of the bubbles. Most of the studies are based on the effects of operating parameters on bubble size reduction. A few studies showed the impact of FC-1100 on bubble size and burst mechanism [1, 14, 15]. According to them, in the absence of a surfactant, bubble size has a pronounced negative relationship with the amount of acid mist produced, with more giant bubbles producing less acid mist. However, in the presence of a surfactant, the influence of bubble size on acid mist is insignificant or unknown [1]. Another study by the same group revealed that more than 54,000 bubbles were sampled and quantified using high-speed cameras. They found that FC-1100 in the solution reduced the average bubble diameter by 9.3 μm [4]. However, the sole mechanism behind the bubble burst is still poorly understood.

Understanding bubble burst dynamics is critical for optimizing surfactants in copper electrowinning. Recent but few research studies have shown that bubble size, liquid viscosity, and surface tension influence the size and velocity of the droplets that form [16]. High-speed imaging techniques have shown that smaller bubbles produce fewer but faster droplets, whereas more giant bubbles produce a more substantial number of smaller droplets [17, 18]. However, there is a serious need to understand the bubble-bursting dynamics. To achieve this, we have proposed a novel methodology: inject a bubble into the copper electrolyte medium and observe its formation, propagation, and bursting at the surface by High-Speed Imaging.

This paper aims to understand the effect of different surfactants on the bubble size and burst mechanism. It looks at the physical–chemical properties of the electrolyte medium, such as density, surface tension, and viscosity, and particularly the impact of surfactants on these properties. We have investigated the average bubble size, which is reduced in the presence of surfactant and significantly reduced in the case of FC-1100. Surfactants reduce the electrolyte’s surface tension and change the electrolyte’s bubble dynamics. Bubble terminal velocity decreases and increases residence time. Based on the parameters stated above, the bubble flow pattern is Stokes pattern, and is calculated using the Reynolds number; the shape is circular and is calculated by Eotvos and Morton numbers; surface tension forces are dominant around the bubble and are computed using Ohnesorge and Weber number. Bubble kinetic energy is calculated at the liquid–air interface. This kinetic energy changes the bubble’s bursting dynamics by being transferred entirely to the bubble’s film cap, which absorbs the impact and results in less energy being used to produce droplets. Here, FC-1100 stands out in producing the smallest bubble size and eventually lesser acid mist droplets. Later, we evaluated all the surfactants at a 20 mg/l concentration for acid mist concentration measurement in a lab scale setup that mimics an industrial electrowinning facility. FC-1100 suppressed the acid mist up to 91.9%. This study reveals that surfactant modifies bubble propagation and bursting dynamics and eventually helps generate smaller droplets. Our work is novel as compared to the previous studies. We have focused understanding of bubble growth, propagation and bursting mechanism in the absence and presence of surfactants with emphasis on surface tension, bubble terminal velocity, residence time and Kinetic energy, that previous studies neglected.

## Experimental

### Materials

A syringe pump and a needle of 10 μm inner diameter was purchased from NewEra and World Precision Instruments, respectively. Five surfactants were used in this study, including FC-1100. For the other four surfactants, we have used made-up names to facilitate discussion, as some of the commercial products are not yet available on the market. We used DAK-1, DAK-2, DAK-3, and DAK-4 to represent these four chemical surfactants. All the surfactants’ properties are provided in Table 1. An electrolyte with 40 g/L Cu and 180 g/L H_2_SO_4_ was used in the experiments. The concentration of the surfactants used in the experiments is kept at 20 mg/L as the optimized concentration for FC1100 mimics the industrial standard according to 3 M. The tests were performed at room temperature.

**Table 1:** Surfactants and their properties used for the experiments

Surfactant name	Concentration (mg/L)	Surface tension (N/m)	Density (Kg/m^3^)	Dynamic viscosity (Kg/m.s)	Impact velocity (*U*_b_) (mm/s)	Critical film thickness (*h*_c_) (m)
FC-1100 (L1)	20	0.021	1455	2.42	3.0	4.13E-50
DAK-1 (L2)	20	0.028	1468	2.42	1.8	1.28E-49
DAK-2 (L3)	20	0.031	1476	2.40	1.5	1.61E-49
DAK-3 (L4)	20	0.024	1502	2.44	2.6	7.93E-50
DAK-4 (L5)	20	0.031	1461	2.37	2.0	1.43E-49
Electrolyte (E1)	0	0.056	1480	2.60	2.1	4.86E-49

### Characterization

The surface tension of the samples was measured by a Rame-Hart tensiometer model 90, which was calibrated using water. The dynamic and kinetic viscosities of the samples were measured using an Ubbelohde capillary viscometer. The constant value for a viscometer at room temperature is 0.002689. All the videos and images are processed using ImageJ software.

### High-Speed Imaging of Bubble

**Fig. 1:**
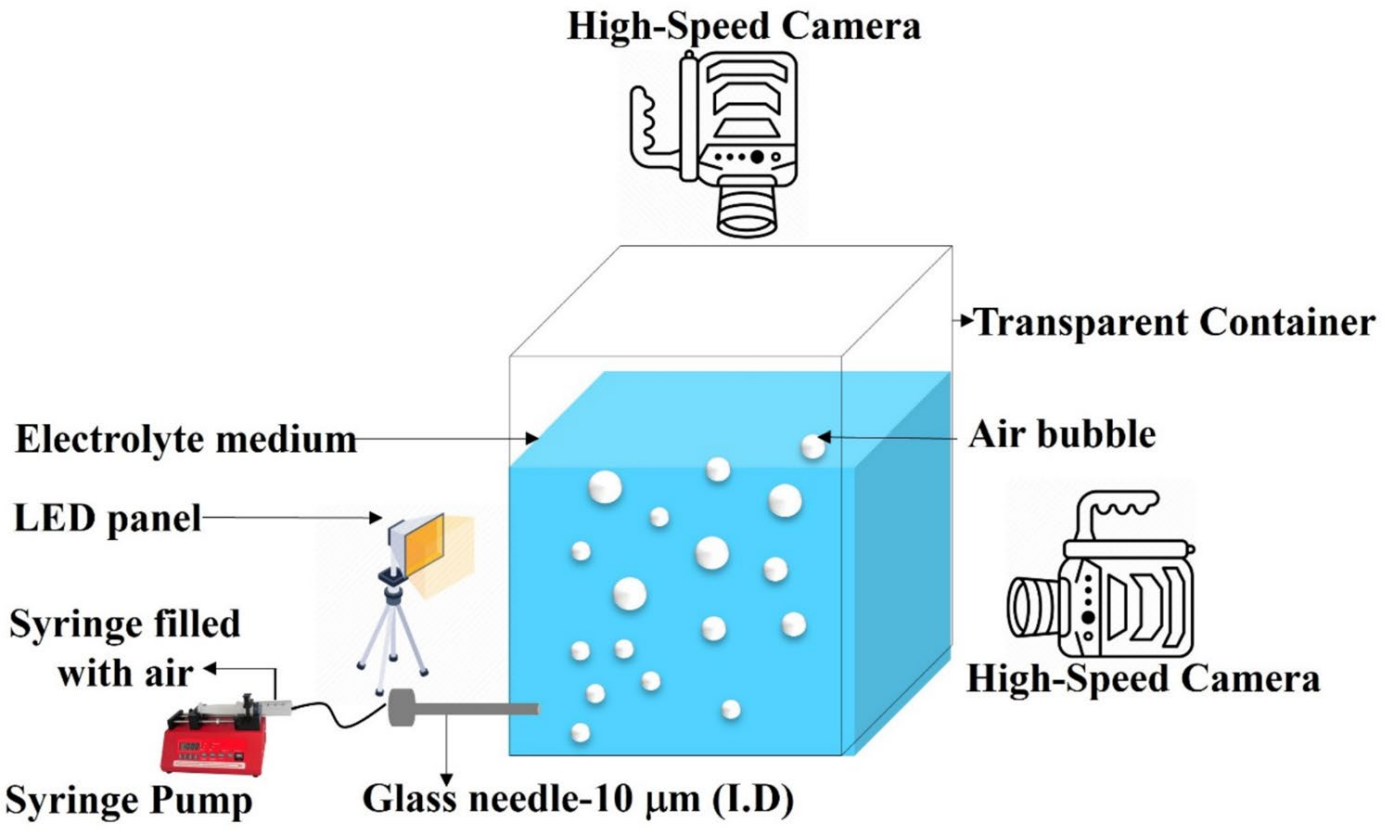
The experimental setup and its essential components for bubble generation

To generate a single bubble in the aqueous medium, a cuboidal polycarbonate column (length = 26.5 cm, breadth = 16.5 cm and thickness = 0.5 cm) with a thick-walled glass capillary (inner diameter dc = 10 μm) kept at the bottom, and a NewEra syringe pump was used. The syringe pump was used to inject air through a needle with an inner diameter of 10 μm into 1 L electrolyte solution. The needle tip was positioned 3 cm below the surface of the electrolyte. A constant flow rate of 25 ml/hr was used to achieve the bubble formation. A Phantom high-speed camera was used to visualize the formation of bubbles at the tip of the capillary, their propagation in the electrolyte as well as bursting at the liquid–air interface. High-speed videos were recorded at two angles: one in front of the solution tank to capture the bubble formation and propagation and another from above the solution to observe bubble coalescence and bursting (Fig. 1). The videos and photos were captured at 7000–9000 frames per second, and the resolution of 1080*1920 was maintained throughout the study. A needle with an outer diameter of 200 μm was used as a reference scale for bubble diameter measurement. ImageJ open-source software was used to evaluate the size of the bubbles, as we have a known reference needle of 200 μm. We first converted the needle’s size into pixels, then measured the bubbles in pixels, and finally converted those measurements back into micrometers (µm) using the reference. All the bubble diameter measurements were the average measurements of the same bubble at 16 different video frames. Bubble diameter, terminal velocity, and residence time were calculated based on the video recordings (See Fig. 4).

### Acid Mist Measurement Chamber

**Fig. 2:**
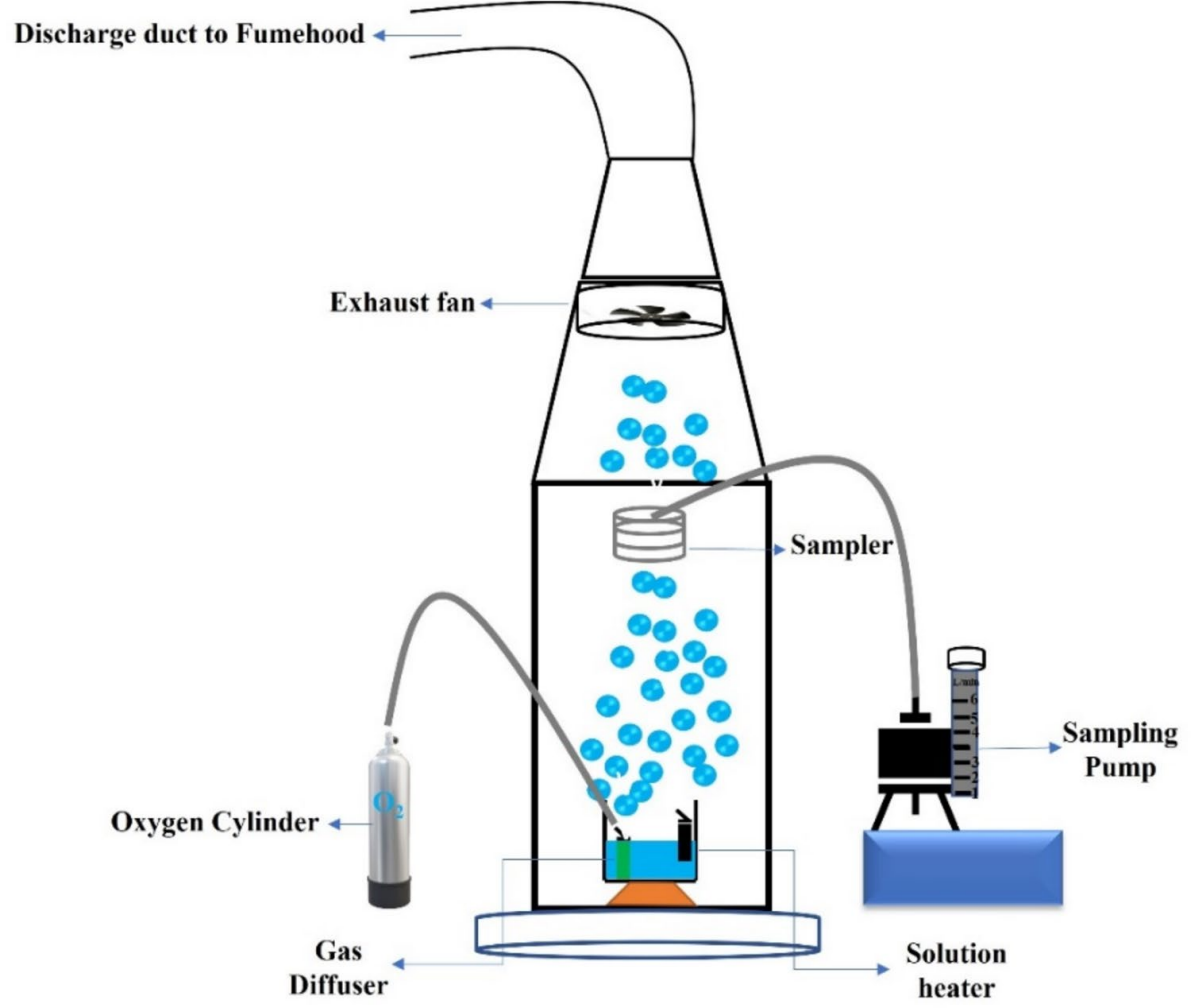
Schematic setup of the chamber used for the acid mist experiment

As shown schematically in Fig. 2, a testing chamber was used to measure the acid mist generation by bubbling oxygen into an electrolyte. The cross-section of the chamber is 50 cm × 50 cm and a height of 1.5 m. Air enters the chamber from the bottom, and a 4-inch diameter exhaust fan at the end of the chamber duct pulls air outward with an air velocity of 2.38 m/s, corresponding to a flow rate of 0.01929 m^3^/s. As the main body of the chamber’s cross-sectional size is 0.5 m × 0.5 m, the flow velocity in the chamber is 0.077 m/s. A polypropylene tank (31.8 × 16.5 × 22.2 cm) is placed on top of a perforated plate that contains 5 L of Cu 40g/L and H_2_SO_4_ 180 g/L electrolyte. The electrolyte temperature was thermostatically controlled at 40 °C by a water heater. During sampling, bubbles were generated by discharging oxygen gas through a submerged gas diffuser in the electrolyte bath [19]. The gas was discharged at a flow rate of 1.5 L/min, regulated by an oxygen flow controller. A total of 6 samples were prepared for the study; one containing only the electrolyte and five others with surfactants added at a concentration of 20 mg/L. All the specifications are mentioned in Table 1. Acid mist tests were performed using 5 L of electrolyte solution.

As oxygen was infused into the solution, bubbles were generated and traveled to the surface/air interface. These bubbles burst at the electrolyte-air interface, emitting droplets of acidic aerosols (acid mist) into the air within the experimental chamber. The droplets of acidic concentration were transported to the sampling point, where air samples were collected using an open-faced sampling cassette equipped with a 37 mm diameter quartz fiber filter (Whatman Q.M.A.). Each sample was collected over 30 min at a sampling rate of 5 L/min to ensure that a sufficient number of vaporized aerosols was captured by the quartz fiber filter.

After samples were collected, the filters were removed from the sampling cassettes and immersed in a 50 mL volumetric flask with deionized water, which was sonicated for 15 min. The sonication process agitated the deionized water to wash off all the acid absorbed on the filter. Atomic absorption spectroscopy was used to determine the acid content in the solution, and calculations were then conducted to find the acid mist concentration at the sampling point within chamber. In this study, cupric sulfate pentahydrate (CuSO_4_·5H₂O) was used as a source of Cu2⁺ ions, which undergo a stoichiometric reaction with sulfuric acid to form copper sulfate (CuSO₄). By measuring the concentration of copper ions in the resulting solution using AAS, and assuming a 1:1 molar reaction ratio between Cu2⁺ and H₂SO₄, the amount of sulfuric acid consumed during the reaction can be inferred. This indirect approach is valid under the assumption that sulfuric acid is the limiting reagent and that copper ions are solely derived from the dissociation of copper sulfate without interference from side reactions. The AAS results provide a quantitative basis for calculating the initial sulfuric acid concentration, making this technique a viable method for sulfuric acid estimation in systems involving known stoichiometric interactions with metal ions.

## Results

A bubble has three stages in total before bursting at the liquid/air interface: (a) Bubble formation, (b) Bubble propagation, and (c) Bursting. All these stages change with the type of liquid. In this case, we have known liquid parameters mentioned in Tables 1 and 2.

**Table 2:** Hydrodynamic and energetic characteristics of bubble in electrolyte

Surfactant	Bubble diameter (μm)	Terminal velocity (*V*_.*T*._) (mm/s)	Residence time (*T*_*.R*._) (sec)	Bubble energy (*E*_.*K*._) (J)	Bubble movies	Reynolds number R_e_	Weber number (W_b_)	Ohnesorge number (O_h_)	Morton number Log 10(M_o_)	Eotvos number (E_o_)
FC-1100	172	9.6	3.11	4.52797E-12	M-1	0.99	^0.001^	0.032	4.9	0.01
DAK-1	322	34	0.86	1.35186E-11	M-2	6.91	0.020	0.020	4.6	0.05
DAK-2	331	36	0.82	1.13151E-11	M-3	7.41	0.020	0.019	4.5	0.05
DAK-3	269	24	1.24	1.28528E-11	M-4	3.99	0.009	0.024	4.9	0.04
DAK-4	285	26	1.12	1.35042E-11	M-5	4.56	0.009	0.021	4.5	0.03
Electrolyte	306	28	1.04	1.30742E-11	M-6	5.01	0.006	0.016	4.9	0.02

### Bubble Formation

**Fig. 3:**
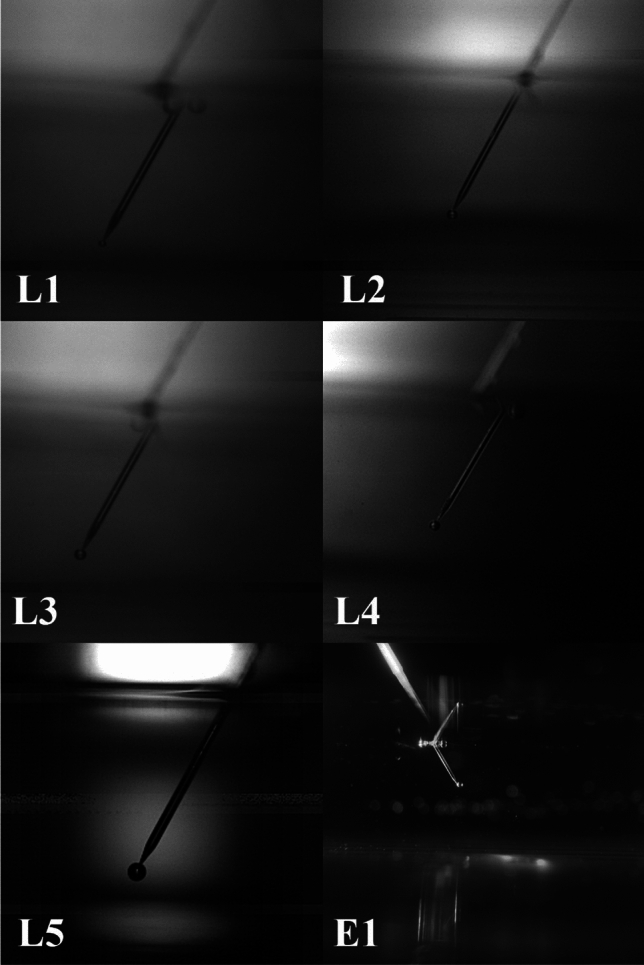
Bubble detachment diameter in different liquid samples

A bubble formation is shown in Fig. 3 and can be seen in the movie names provided in Table 2 and files are in supplementary file. The bubble detachment diameter from the needle tip was measured at 40 ms after formation for all samples. The respective bubble diameters are mentioned in Table 2. In the presence of bare electrolytes, the bubble diameter was 306 μm, and in the presence of surfactants, the bubble diameter was reduced except for DAK-1 and DAK-2. FC-Table 1.

### Bubble Propagation

**Fig. 4:**
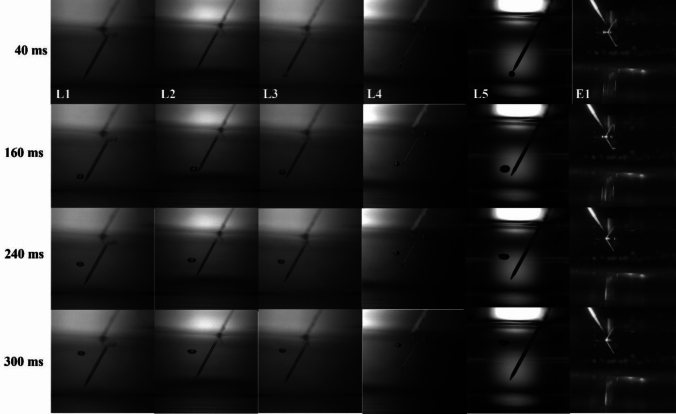
Bubble progression over time for tested surfactant solutions (surfactant names are shown in the top row)

Bubbles in bare electrolyte move up abruptly in the liquid due to higher Reynolds number and more air volume, leading to faster bursting and droplet production. However, in the presence of FC-1100, bubbles rise more slowly, have longer residence times, and exhibit different bursting dynamics due to a lower Reynolds number and increased time for surface stabilization. As the bubble detached from the needle tip, it propagated with its local velocities and reached its terminal velocity, as mentioned in Table 2. If a bubble is larger, it has more air volume but less pressure, and vice versa. The pressure inside a bubble is proportional to the surface tension of the liquid and the radius of the bubble. Equation 2 explains the link between pressure and a bubble’s size. Smaller bubbles will move slower than bigger ones and have more residence time in liquid. Here, FC-1100 has the lowest terminal velocity as its bubble diameter is smaller than others. In the case of FC-1100, the bubble has more liquid residence time and a delayed bursting time. However, in bare electrolyte, the residence time was 1.04 s, while the addition of FC1100 increased the residence time to 3.11 s. Other surfactants have less impact on residence time. Bubbles in bare electrolyte move abruptly into the liquid because of a higher Reynolds number (c.f. Table 2). The Reynolds number is calculated using Eq. 7. A bubble with a larger air volume has a higher chance of bursting quickly and eventually producing droplets. In the case of FC-1100, the bubble rises more slowly because of the lower Reynolds number, and its bursting dynamics change as it has more time to stabilize at the electrolyte-air interface. Figure 4 provides a visual understanding of a bubble’s progression over time. The time scale in Fig. 4 begins at 40 ms, representing the moment the bubble detaches. It continues until the bubble reaches the surface and eventually bursts at 300 ms.

### Bubble Bursting

When a bubble bursts at the liquid/air interface, it bursts into film and jet drops predominantly. Figure 6 illustrates that when a bubble reaches its critical film thickness, it starts to burst. The critical film thickness can be calculated using the formula in Eq. 1 and the values are summarized in Table 2.

1 $$ h_{c} \, = \,0.267\,\left( {\frac{{{\text{a}}_{{\text{f}}} * {\text{A}}_{{\text{H}}}^{2} }}{6\pi \sigma \Delta P}} \right)^{\frac{1}{7}} $$

2 $$ \Delta P\, = \,\frac{4\sigma }{r} $$

where *a*_f_ is the area of the film, A_.H._ is Hamaker constant, Δ*P* is differential pressure, *σ* is the surface tension, and *π* is 3.14.

Bubble formation, bubble propagation through the liquid, and bubble bursting dynamics can be understood with its terminal velocity (*V*_.T._) and residence time (*T*_R_). Terminal velocity dictates how fast the bubble reaches the bursting surface. Residence time (*T*_.R._) dictates how much time the bubble resides in the liquid before bursting and its propagation in the liquid with Reynolds number. A bubble’s terminal velocity directly correlates with bubble diameter and the liquid’s dynamic viscosity. Bubble terminal velocity (m/s) and residence time (*s*) can be calculated using Eqs. 3 and 4.

3 $$ V_{T} \, = \,\frac{2}{9}\,\frac{{\left( {\rho_{{{\text{liquid}}}} - \rho_{{{\text{bubble}}}} } \right)\,gr^{2} }}{{\mu_{{{\text{liquid}}}} }} $$

4 $$ T_{R} \, = \,\frac{L}{{V_{T} }} $$

where *µ*_liquid_ is the dynamic viscosity of the liquid, *ρ*_liquid_ is the liquid density, *ρ*_bubble_ is the bubble air density, *g* is the acceleration due to gravity, *r* is the bubble radius, *V*_.T._ is the terminal velocity and *L* is the column height.

**Fig. 5:**
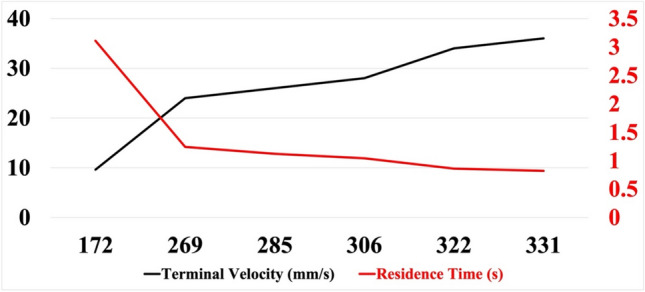
Terminal velocity and Residence time profiles change with bubble diameters

Figure 5 depicts the terminal velocities (m/s) and residence time (s) profile of bubbles rising indifferent test solutions. Terminal velocity increases with bubble diameter, and thus, residence time decreases. Smaller bubbles have less air volume, and bigger bubbles have more air volume, accelerating the bubble. Bubbles accelerated quickly after detachment and reached terminal velocity when the force of buoyancy and force of gravity balanced each other from the capillary opening. At the time of detachment, the bubble appeared nearly spherical (c.f. Figure 3), a shape later confirmed in the discussion section using the Morton and Eotvos numbers. However, distortions gradually grew until a steady-state spherical shape was produced at the terminal velocity distance.

Bubbles with higher terminal velocities have lower residence times, which eventually depend on the bubble shape and its flow dynamics in the liquid [20]. The bubble’s shape was nearly spherical for our study, as seen in Fig. 3. Morton (M_o_) and Eotvos (E_o_) are dimensionless numbers that help us understand the bubble shape, and they are calculated using Eqs. 5–7. Once the bubble shape is known, the bubble’s flow dynamics become imperative as the bubble must flow through the fluid. Reynold’s number helps to understand the bubble’s flow dynamics in the fluid. Reynolds number is smaller than 1 for FC1100 and within 1–10 for other surfactants. If this number is much smaller than 1, it indicates that surface tension forces dominate over viscous and inertial forces. As a result, the bubble tends to maintain a stable, spherical shape, resisting deformation and minimizing its surface area. In the case of FC1100, Reynold’s number for the liquid sample was 0.99, miming the stokes flow and creeping flow patterns in the fluid samples [21]. This states that bubble propagation will be smooth and reach the surface very slowly, which is why, unlike others, it has more residence time and less terminal velocity. Additionally, FC1100 has the lowest surface tension value among all the surfactants, which could also explain the bubble’s high residence time and low terminal velocities.

5 $$ M_{o} \, = \,\frac{{\left( {\rho_{{{\text{liquid}}}} - \rho_{{{\text{bubble}}}} } \right) * g * \mu_{{{\text{liquid}}}}^{4} }}{{\sigma^{3} \rho_{{{\text{liquid}}}}^{2} }} $$

6 $$ E_{o} \, = \,\frac{{g * d^{2} \left( {\rho_{{{\text{liquid}}}} - \rho_{{{\text{bubble}}}} } \right)}}{\sigma } $$

7 $$ R_{e} \, = \,\frac{{\rho_{{{\text{liquid}}}} * V_{T} * d}}{{\mu_{{{\text{liquid}}}} }} $$

where *µ*_liquid_ is the dynamic viscosity of the liquid, *ρ*_liquid_ is the liquid density, *ρ*_bubble_ is the bubble air density, *g* is the acceleration due to gravity, *d* is the bubble diameter, *V*_.*T*._ is the terminal velocity and *σ* is the surface tension.

According to the analysis, surface tension forces mostly determine bubble shape and stability in the solution. Weber and Ohnesorge’s numbers reveal that there is little deformation because surface tension strongly dominates inertial and viscous forces. However, based on the *Mₒ* and *Eₒ* numbers, the bubble shape is confirmed to be spherical. The *R*ₑ indicates that surface tension forces are dominant in the FC1100 sample, while for the other liquid samples, *Rₑ* falls between 1 and 10. This means viscous forces dominate, and inertial forces begin to play a minor role. In such a case, the bubble’s flow will be smooth, but the fluid may start to experience changes due to inertial forces. However, bubble propagation dynamics get more complex because of fluid dynamic viscosity. For a more comprehensive understanding of bubble dynamics, the discussion on Weber’s number and Ohnesorge’s number becomes critical. Weber and Ohnesorge’s numbers are smaller than 1 for all liquid samples. The combined effect of Weber and Ohnesorge’s number states that surface tension forces are dominant over inertial and viscous forces[22]. Both these numbers are much smaller than 1, meaning surface tension forces dominate. This means that the bubble will propagate smoothly and steadily with minimal deformation. Equations 8 and 9 are used to calculate the Weber and Ohnesorge numbers.

8 $$ W_{e} \, = \,\frac{{\rho_{{{\text{liquid}}}} * V_{T}^{2} * d}}{\sigma } $$

9 $$ Oh\, = \,\frac{{\mu_{{{\text{liquid}}}} }}{{\sqrt {\rho_{{{\text{liquid}}}} * \sigma * d} }} $$

where *µ*_liquid_ is the dynamic viscosity of the liquid, *ρ*_liquid_ is the liquid density, *V*_.T._ is the terminal velocity, *σ* is the surface tension, and *d* is the bubble diameter.

**Fig. 6:**
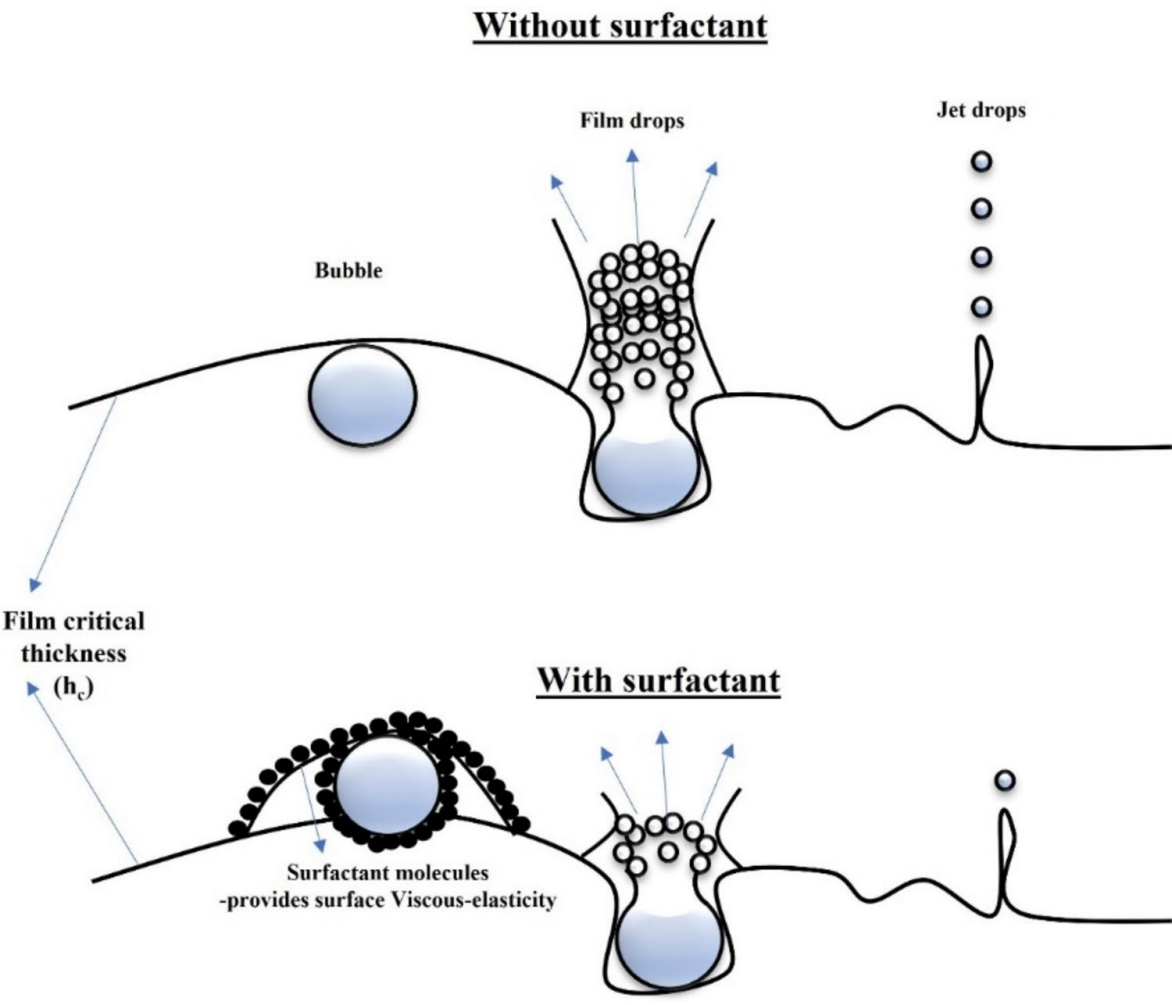
Bubble bursting phenomena with and without surfactant

The Bubble’s potential and kinetic energy becomes imperative when the bubble reaches the surface. This potential and kinetic energy helps in bubble bursting. With M_o_, R_e_, E_o_, W_e_, and O_h_ discussed above. It is confirmed that when a bubble detaches from the needle orifice, it gets spherical shape, and on that bubble, surface tension forces are dominant. However, different bubbles have different potential energy during propagation based on their diameters until they reach the liquids’ surface [23]. This stored potential energy converts into Kinetic energy when the bubble’s shape is deformed as it reaches the surface. As illustrated in Fig. 6, as the bubble reaches the surface, it forms a film over the liquid surface because of the surfactant’s visco-elastic behavior. The addition of the surfactants makes the liquid film Visco-elastic, which is necessary for the dynamic stabilization of the liquid film, and later, it stretches to its critical film thickness before bursting [24].

The bubble’s surface tension and kinetic energy are interrelated. Kinetic energy overcomes the effect of surface tension to burst the bubble at the surface. The bubble experiences changes in the surface tension and buoyancy as it reaches the surface, and the bubble starts to experience deformation as buoyancy pushes it upward and the surface tension stretches the bubble inward. When the buoyancy force overcomes the surface tension, the bubble bursts [25]. The bubble’s kinetic energy (*E*_k_) is transferred first to the liquid film. Then, most of the kinetic energy is utilized to reach the critical thickness of the liquid film (c.f. Table 2). During bursting, the bubble possesses very little or negligible kinetic energy just before it ruptures (Fig. 6). This eventually changes its bursting impact and dynamics. As a result, less film and jet drops are produced during the bubble’s rupture [1, 26]. The bubble critical thickness and the bubble kinetic energy of collision with the liquid interface can be calculated using Eqs. 1 and 8, respectively [27]. All the values for critical film thickness and bubble kinetic energy are summarized in Table 2. However, the interesting phenomenon is that bubble’s kinetic energy and bubble film thickness have not drastically changed for all the liquid samples.

Bubble kinetic energy for all the samples is enough to overcome the bubble critical film thickness. However, the bursting dynamics have changed because of the 50% reduction in bubble size, especially for FC-1100, compared to E1 and other samples mentioned in Table 2, and its interaction with different surfactants because smaller bubbles have less air during bursting. The critical parameters here are surface tension and Reynolds number of all the liquid samples. Specifically, FC-1100 has the lowest surface tension and Reynolds number, which is less than 1. This shifts the bubble in the stokes flow regime and confirms that the bursting dynamics have changed for the bubble with FC-1100 surfactant and eventually has less impact on jet and film drops [21].

10 $$ E_{k} \, = \,0.5 * C_{m} * \rho_{{{\text{liquid}}}} * V_{b} * U_{b} $$

11 $$ C_{m} \, = \,0.62 * \chi - 0.12 $$

12 $$ \chi = \frac{{d_{{\text{H}}} }}{{d_{{\text{V}}} }} $$

where *ρ*_liquid_ is the liquid density, *V*_b_ is the bubble volume, *U*_b_ is bubble impact velocity, *C*_*m*_ is the added mass coefficient as a function of bubble deformation ratio, and *χ* is the ratio of horizontal (*d*_H_) and vertical (*d*_V_) bubble diameter.

**Fig. 7:**
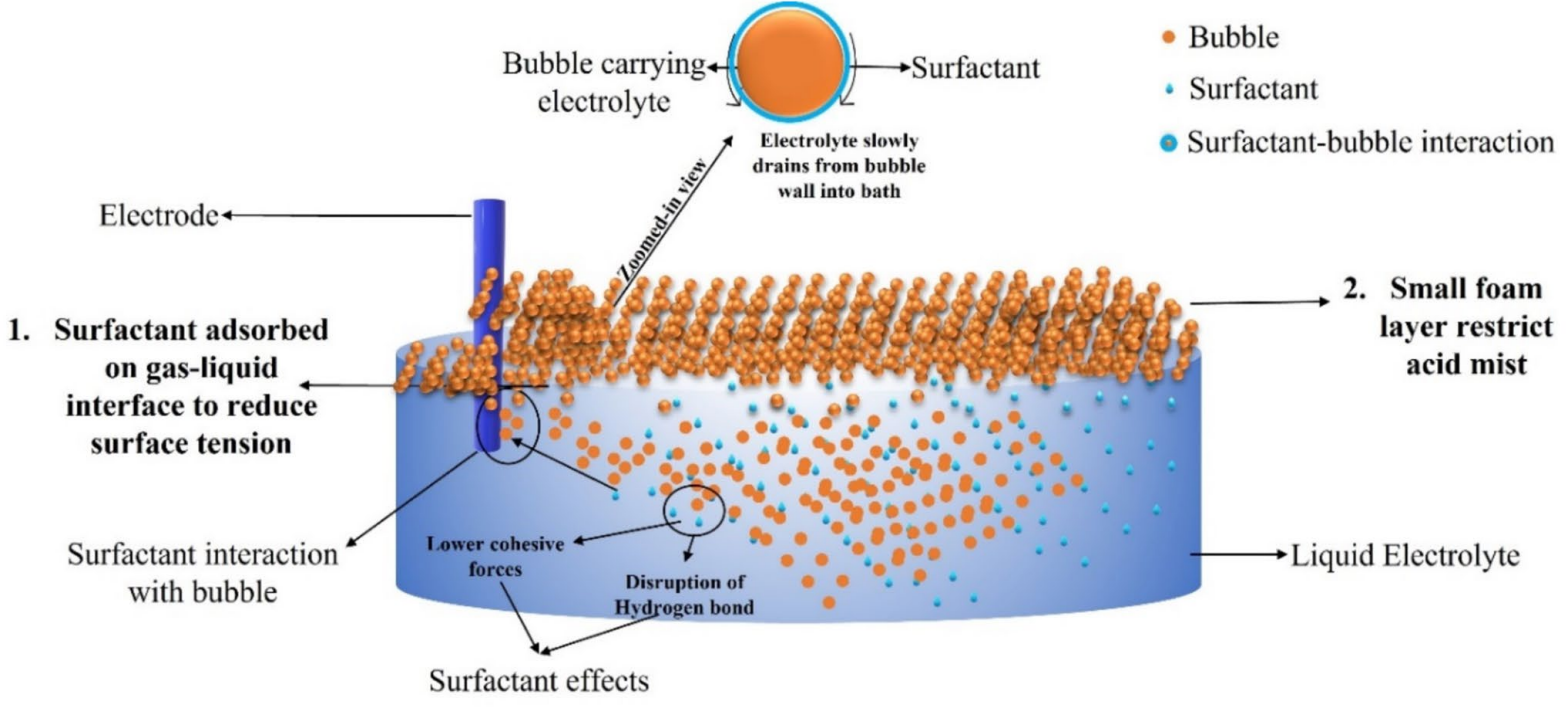
Surfactant working mechanism in acidic medium

Surfactants reduce the surface tension of the liquid and eventually reduce the bubble diameter. A smaller bubble diameter, resulting from a liquid with lower surface tension also affects the bubble’s propagation and bursting dynamics. Eventually, it changes the bubble’s terminal velocity and residence time. Figure 7 illustrates the working mechanism of the surfactant with the liquid samples. Surfactants adsorb on the bubble-liquids interface, gradually reducing bubble growth and promoting liquid drainage from the bubble film. In some cases, it also forms a foam layer over the liquid. However, foaming for some applications, such as electrowinning, is not beneficial. For FC-1100 surfactant, the bubble’s residence time in the liquid was maximum, i.e., 3.11 s, and has the minimum terminal velocity, i.e., 9.6 mm/s, which gives the bubble more time to stabilize and reach stokes flow regime [21]. FC-1100 has been bestowed among all other surfactants regarding bubble size and its bursting dynamics at the interface.

### Acid Mist Experiments

**Fig. 8:**
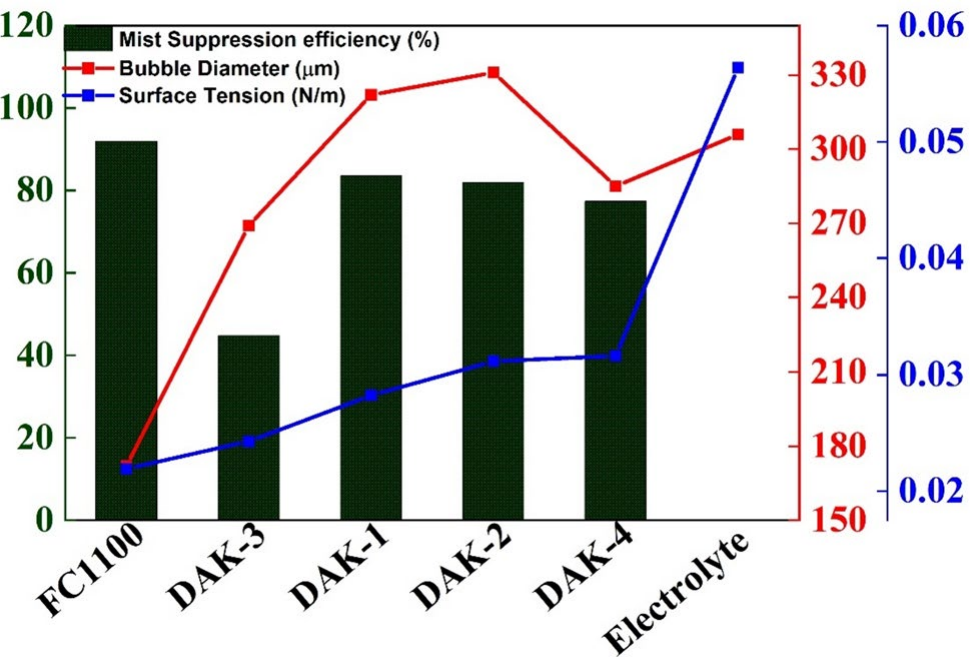
Surfactant’s effect on acid mist suppression, bubble diameter and surface tension

Our study aims to understand the effect of different surfactants on the bubble size, terminal velocity, residence time and burst mechanism. Figure 8 illustrates the results of acid mist suppression efficiency in the presence of different surfactants and compares the suppression efficiency with residence time, terminal velocity, bubble diameter and surface tension. Results revealed that acid mist suppression efficiency is inversely related to bubble diameter and surface tension. Bubble’s terminal velocity and residence time also correlates with suppression efficiency. Our studies confirm FC-1100 has maximum acid mist suppression efficiency because it alters the bubble diameter, terminal velocity and residence time, surface tension of the liquid, and its bursting dynamics at the liquid–air interface. Terminal velocity, residence time, Weber number and Ohnesorge number follow the same trend as acid mist suppression. However, DAK-3 deviates from this pattern, as it promotes bubble coalescence rather than suppression (c.f. movie M-4 in supplementary file). Particularly for FC1100 Reynolds number is less than 1, which aids bubble stabilization and reaches Stokes flow before bursting [21, 28]. One unstated reason could be that FC1100 is a fluorinated surfactant with high electronegative fluorine atoms. Because of fluorine’s high electronegativity, low surface energy, and polar nature, fluorine atoms migrate to the air–water interface on empty bubble sites. Fluorine atoms improve the surfactant film’s rigidity and stability at the contact. This enhanced stability prevents bubble coalescence, allowing for smaller bubble diameters. In comparison to their hydrocarbon counterparts, fluorinated surfactants create micelles faster. These micelles can trap and stabilize gas molecules, forming smaller and more uniform bubbles. FC1100 can repel smaller bubbles from each other at the bubble surface. This electrostatic repulsion also reduces bubble coalescence and aids in maintaining smaller bubble diameters. All the surfactants follow the same trend, however, DAK-3 has a bubble diameter smaller than other surfactants, but its suppression efficiency is not at par with others. One of the possible reasons for this anomaly is the bubble coalescence and bubble instability. In Table 2, movie (M-4), visually shows the bubble coalescence and makes the bubble unstable.

Our study shows that with the use of surfactants, the electrolyte’s surface tension decreases and alters the bubble diameter, terminal velocity and residence time, surface tension of the liquid film, and its bursting dynamics at the liquid–air interface (Fig. 8). The surfactants evaluated in this study are widely accepted in copper electrowinning. With the use of surfactants, film and jet drops responsible for sulfuric acid mist are reduced as the bubble’s bursting impact is less. However, not all surfactants have the same impact as FC-1100. Hence to understand this issue, we would evaluate further to understand electrolyte’s surface dilatational viscosity.

## Conclusion

In this work, we have investigated the effect of surfactants on bubble size and their bursting dynamics. The study shows that surfactants reduced the surface tension and bubble size; bubble-bursting dynamics were altered accordingly. The maximum surface tension and bubble size reduction were seen for FC1100. The bubble-bursting dynamics were studied using a high-speed camera, and the results show that bubble size and electrolyte’s surface tension decreases with the addition of surfactant. FC-1100 had the highest impact on bubble size and surface tension as compared to other surfactants. As a result of that bubble’s flow dynamics, residence time and terminal velocity in the electrolyte changed. FC-1100 had the lowest bubble terminal velocity, and the highest residence time. This gave the bubble more time to stabilize in the liquids, assume a spherical shape, and reach its kinetic energy. That extra kinetic energy was transferred to the bubble film during bursting, altering its bursting dynamics. The kinetic energy was used to stretch the bubble film, with most of it contributing to the bubble’s rupture at the interface. Because of the sudden change in surface tension at the interface, the jet and film drop have less energy and less impact during bubble bursting. To confirm our results, we have evaluated the surfactants for real-time sulfuric acid mist suppression in a chamber that simulates the bubble formation during the copper electrowinning process. The results show that FC1100 has the maximum sulfuric mist suppression efficiency compared to others because of its lower surface tension and smaller bubble size. The limitation of the study is that the bubble high-speed video imaging tests were done on a single bubble generation but not during the electrowinning process. Observing and studying thousands of bubbles during the electrowinning process was extremely hard. However, this study advances knowledge of the working mechanism of the surfactants. The potential future research direction is to observe and study the bubbles in real electrowinning plants.
